# Establishment of an antibody specific for AMIGO2 improves immunohistochemical evaluation of liver metastases and clinical outcomes in patients with colorectal cancer

**DOI:** 10.1186/s13000-021-01176-2

**Published:** 2022-01-30

**Authors:** Keisuke Goto, Mitsuhiko Osaki, Runa Izutsu, Hiroshi Tanaka, Ryo Sasaki, Akimitsu Tanio, Hiroyuki Satofuka, Yasuhiro Kazuki, Manabu Yamamoto, Hiroyuki Kugoh, Hisao Ito, Mitsuo Oshimura, Yoshiyuki Fujiwara, Futoshi Okada

**Affiliations:** 1 Division of Experimental Pathology, Tottori University Faculty of Medicine, 86 Nishicho, 683-8503 Yonago, Japan; 2 Division of Gastrointestinal and Pediatric Surgery, Department of Surgery, School of Medicine, Tottori University Faculty of Medicine, 683-8503 Yonago, Japan; 3 Chromosome Engineering Research Center (CERC), Tottori University, 683-8503 Yonago, Japan; 4 Trans Chromosomics, Inc., 683-8503 Yonago, Tottori, Japan; 5 Division of Genome and Cellular Functions, Tottori University Faculty of Medicine, 683-8503 Yonago, Japan

**Keywords:** AMIGO2, Monoclonal antibody, Colorectal cancer, Liver metastasis, Prognosis

## Abstract

**Instruction:**

The human amphoterin-induced gene and open reading frame (AMIGO) was identified as a novel cell adhesion molecule of type I transmembrane protein. AMIGO2 is one of three members of the AMIGO family (AMIGO1, 2, and 3), and the similarity between them is approximately 40% at the amino acid level. We have previously shown that AMIGO2 functions as a driver of liver metastasis. Immunohistochemical analysis of AMIGO2 expression in colorectal cancer (CRC) using a commercially available anti-AMIGO2 mouse monoclonal antibody clone sc-373699 (sc mAb) correlated with liver metastasis and poor prognosis. However, the sc mAb was found to be cross-reactive with all three molecules in the AMIGO family.

**Methods:**

We generated a rat monoclonal antibody clone rTNK1A0012 (rTNK mAb) for human AMIGO2. The rTNK mAb was used to re-evaluate the association between AMIGO2 expression and liver metastases/clinical outcomes using the same CRC tissue samples previously reported with sc mAb.

**Results:**

Western blot analysis revealed that a rTNK mAb was identified as being specific for AMIGO2 protein and did not cross-react with AMIGO1 and AMIGO3. The rTNK mAb and sc mAb showed higher AMIGO2 expression, which correlates with a high frequency of liver metastases (65.3% and 47.5%, respectively), while multivariate analysis showed that AMIGO2 expression was an independent prognostic factor for liver metastases (*p* = 7.930E-10 and *p* = 1.707E-5). The Kaplan-Meier analyses showed that the rTNK mAb (*p* = 0.004), but not sc mAb (*p* = 0.107), predicted worse overall survival in patients with high AMIGO2 expression. The relationship between AMIGO2 expression and poor disease-specific survival showed a higher level of significance for rTNK mAb (*p* = 0.00004) compared to sc mAb (*p* = 0.001).

**Conclusions:**

These results indicate that the developed rTNK1A0012 mAb is an antibody that specifically recognizes AMIGO2 by immunohistochemistry and can be a more reliable and applicable method for the diagnostic detection of liver metastases and worse prognosis in patients with high AMIGO2-expressing CRC.

**Supplementary Information:**

The online version contains supplementary material available at 10.1186/s13000-021-01176-2.

## Introduction

Cancer survival has improved globally over the last two decades, with an increase in the survival of patients with deadly cancers, such as liver, pancreatic, and lung, by up to 5% [1]. The main cause of reduced survival in cancer patients and survivors is distant metastasis rather than growth of the primary tumor, accounting for approximately 90% of cancer-related deaths [2–4]. Of the common organs that cause distant metastases, the liver is the most frequent site (59%), except for regional lymph nodes [5]. The precise mechanism of metastasis to distant organs, including the liver, that fundamentally undermines cancer therapeutic strategies, is not well-understood [6]. Therefore, finding diagnostic biomarkers and therapeutic targets that reliably determine liver metastases is an urgent task.

We have identified that AMIGO2, a member of the AMIGO family (AMIGO1, 2, and 3), functions as a driver gene for liver metastasis in a mouse model [7]. The AMIGO protein was originally identified as a novel cell adhesion molecule that accompanies type I transmembrane proteins containing six leucine-rich repeats and one immunoglobulin-like domain in the extracellular region that are preferentially expressed on fiber tracts of neuronal tissues which participate in axon tract development [8]. The three AMIGO family molecules are structurally similar and have shown homophilic and heterophilic binding mechanisms among their molecules; however, the organ expression pattern and biological functions of each protein are independent [9]. We found that knocking down AMIGO2 expression, which is highly expressed in liver metastatic tumor cells, reduces the adhesion of tumor cells to hepatic endothelial cells and suppresses liver metastasis. Conversely, it was verified that forced expression of the AMIGO2 gene in non-liver metastatic tumor cells increases the adhesion of tumor cells to hepatic endothelial cells and thereafter forms liver metastases [10]. However, AMIGO2-expressing tumor cells did not show increased adhesion to lung endothelial cells and did not affect lung metastases or metastases to other organs. Thus, it was clarified that tumor cells highly expressing AMIGO2 selectively adhere to hepatic endothelial cells expressing AMIGO family molecules by homophilic/heterophilic adhesion patterns to cause liver metastasis [10].

Extrapolation of this phenomenon in human cancers has been reported by immunohistochemical staining of AMIGO2 expression in human colorectal cancer (CRC) tissue, which is closely associated with AMIGO2 expression and liver metastasis, but not with lung metastasis and peritoneal dissemination [11]. Multivariate analysis showed that AMIGO2 expression in patients with CRC was an independent predictive factor for liver metastasis [11]. Furthermore, transcriptionally high levels of AMIGO2 are associated with shortened survival of patients with CRC [11], breast cancer [12], and gastric cancer [13]. AMIGO2 has been reported as a novel pathogenesis-related gene in gastric cancer [14], melanoma [15, 16], ovarian cancer [17], and pituitary neuroendocrine tumors [18]. In addition, bioinformatics analysis using the transcriptome database selected AMIGO2 as a cancer-related gene candidate for CRC [19], pancreatic cancer [20], and endometrial cancer [21].

Most studies have been conducted on the expression of AMIGO2 mRNA in tumor tissues. However, a high expression of AMIGO2 mRNA has been detected in normal tissues, and is most prominent in the cerebellum, retina, liver, and lung [9]. Lower but steady AMIGO2 expression is also found in the cerebrum, kidneys, small intestine, spleen, and testis [9]. These physiological conditions indicate that as long as AMIGO2 expression is assessed as tissue-wide mRNA expression, its association with carcinogenesis in each organ cannot be determined. Therefore, immunohistochemical staining is the most reliable method for accurately evaluating AMIGO2 expression in cancerous tissues. For this purpose, we have analyzed AMIGO2 expression by using commercially available anti-AMIGO2 mouse monoclonal antibody (sc-373699); however, we noticed that the antibody recognizes AMIGO1 and AMIGO3 as well as AMIGO2. We also investigated the specificity of three commercially available affinity purified polyclonal antibodies. These antibodies were also recognized as AMIGO family molecules.

In this study, we developed a new AMIGO2-specific antibody that does not react with AMIGO1 and AMIGO3, and compared the efficacy of the new antibody using the same tissue samples that previously reported a significant correlation between AMIGO2 expression in primary CRC tissue and liver metastasis [11].

## Materials and methods

### Cell culture and transfection

P3X63Ag8.653 myeloma cells (CRL-1580) and HepG2 hepatocellular carcinoma cells (HB-8065) were purchased from the American Type Culture Collection (ATCC, Manassas, VA). P3X63Ag8.653 cells and HepG2 cells were respectively maintained in RPMI-1640 medium (05911; Nissui Pharmaceutical, Tokyo, Japan) and Dulbecco’s modified Eagle’s medium (05919; Nissui Pharmaceutical) containing 10% heat-inactivated fetal bovine serum (FBS, F7524, Sigma-Aldrich, St. Louis, MO) and L-glutamine. The cell lines were maintained at 37 °C in a humidified 5% CO_2_/95% air mixture.

Expression plasmids for human AMIGO1 (pEZ-M02/AMIGO1), AMIGO2 (pEZ-M02/AMIGO2), and AMIGO3 (pEZ-M02/AMIGO3) were purchased from GeneCopoeia (EX-E1371-M02, EX-E1271-M02, EX-E1133-M02, Rockville, MD). The pEZ-M02 vector was used as the control plasmid. HepG2 cells were transfected with these plasmids using Lipofectamine 2000 reagent (12566014, Thermo Fisher Scientific, Waltham, MA) according to the manufacturer’s instructions. Transfectants stably expressing the introduced vector plasmid were selected by continuous neomycin treatment at 450 µg/mL (10131035, Thermo Fisher Scientific). Neomycin-resistant cells were cloned by the limiting dilution method and maintained in a medium containing neomycin.

### Antigens

To produce an antigen for immunization, a DNA fragment of AMIGO2 extracellular domain (NM_001143668) was amplified by PCR using primers (forward: 5′-GCG AAG CTT GTG TGC CCC ACC GCT TGC AT-3′ and reverse: 5′-GCG CTC GAG TGT GTT AAA TGC CTC ATG AGC ATG GG-3′), and was subcloned into pET-32b(+) (69016, Sigma-Aldrich) using *Hin*dIII and *Xho*I restriction enzymes (resulting in pET32b-AMIGO2-EX). AMIGO2-EX protein was difficult to express in *Escherichia coli* Rosseta-gami B (DE3) pLysS (71137, Merck KGaA, Darmstadt, Germany). pET32b-AMIGO2-EX was digested with *Eco*RI (an *Eco*RI site is located near the upstream end of the leucine-rich repeat sequence), blunted using Blunting high, and then digested with *Eco*RV (at a site upstream of AMIGO2-EX) to eliminate its leucine-rich repeat sequence. Therefore, this vector consisted of the Ig-like domain of AMIGO2 (named pET32b-AMIGO2-Ig) and produces the Trx-AMIGO2-Ig recombinant protein. To obtain the GST-AMIGO2-Ig recombinant fusion protein, pGEX-6P-1 (28954648, GE Healthcare, Chicago, IL) was modified by inserting the synthesized DNA (5′-ACG AGA TCT GCC ATG GAC AAG CTT GTC GAC ACG AGC TCG AAT TCG GAT CCC CCG GGG CTC GAG CAC CAC CAC CAC CAC CAC TGA GCT GAG CGG CCG CTC A-3′) using *Bgl*II and *Not*I restriction enzymes (resulting in pGEX-MCS-His). The amplified AMIGO2-Ig fragment was also cloned into pGEX-MCS-His. Restriction enzymes and DNA-modifying enzymes were purchased from New England Biolabs (Ipswich, MA) and Toyobo (Tokyo, Japan), respectively. Primers were ordered from Eurofins (Huntsville, AL). *E. coli* strain (DH5α) competent cells were obtained from Takara Bio (9057, Shiga, Japan).

After transformation of *E. coli* gami B pLysS (DE3) with each vector, the recombinant proteins were expressed by induction with 1.0 mM isopropyl-β-D (-)-thiogalactopyranoside (094-05144, Fujifilm Wako Pure Chemical, Osaka, Japan) in a LB medium. Transformation using the empty vector, pET-32b(+) was also carried out to produce the Tag protein for use in hybridoma screening as a negative control. After harvesting the cells and sonication, the recombinant proteins were obtained as inclusion bodies. Following solubilization with 6 M guanidine hydrochloride (078-05003, Fujifilm Wako Pure Chemical) in PBS with 0.1 mM glutathione (oxide form, 078-03333, Fujifilm Wako Pure Chemical) and 1 mM glutathione (redox form, 077-02011, Fujifilm Wako Pure Chemical), recombinant protein was purified using Ni-NTA columns (30410, Qiagen, Hulsterweg, Venlo, Netherlands) with elution using 100 mM imidazole (091-00012, Fujifilm Wako Pure Chemical) containing 6 M guanidine hydrochloride. After dialyzing the eluted fraction against PBS containing 0.4 M arginine (PBS-A, 091-04611, Fujifilm Wako Pure Chemical), samples were diluted to about 1 mg/mL and stored at -30 °C.

### Immunization

Protein antigens were prepared in PBS or in PBS-A at 1 mg/mL, and the volume corresponding to the desired amount of protein was increased to an injectable volume with PBS or PBS-A. This volume was then mixed 1:1 (v/v) with either Freund’s adjuvant, complete (F5881, Sigma-Aldrich), or Sigma adjuvant system (S6322, Sigma-Aldrich). For viscous adjuvants, the solution was mixed by repeated passage through a syringe until a smooth emulsion was formed (over approximately 30 min on ice). Injections were performed on 6-week-old male and female Jcl:Wistar rats (Clea, Tokyo, Japan) using a 1 mL glass syringe and a 27-gauge needle. Prime and boost injections of 250 µg protein were injected intraperitoneally every two weeks. Final boosts of 250 µg of protein were delivered intravenously without adjuvant via the tail vein. Volumes varied depending on the injection route and experimental requirements and were in accordance with the relevant JP Home Office animal license for the procedure. The experimental protocol was approved by the Committee of the Institute for Animal Experimentation of Tottori University (17-Y-28).

### Hybridoma generation

After confirming induction of serum antibodies against AMIGO2-Ig protein, spleens and lymph nodes from immunized rats were harvested from euthanized rats, homogenized to single-cell suspensions, and fused with myeloma P3X63Ag8.653 cells using an electro-cell-fusion generator (ECFG21; Nepagene, Chiba, Japan). Fused hybridoma cells were plated in 96-well plates. After approximately 14 days of culture, a primary screen of supernatants was performed by ELISA. Hybridoma clones producing AMIGO2-specific Abs were identified by ELISA using GST-AMIGO2-Ig following HAT selection. Positive wells were picked and passaged in 96-well plates. Each supernatant was reanalyzed by ELISA using Tag, Trx-AMIGO2-Ig, and GST-AMIGO2-Ig. Hybridoma clones that reacted with Trx-AMIGO2-Ig and GST-AMIGO2-Ig, but not Tag, were established using two or more limited dilutions.

### Hybridoma screening

Hybridoma cells producing AMIGO2-specific mAb were identified using ELISA. In brief, a 96-well immunoassay plate (44-2404, Thermo Fisher Scientific) were coated with 50 µg/well antigen, washed three times with PBS-T (0.05% v/v Tween-20: 160-21211, Fujifilm Wako Pure Chemical), and blocked with PBS containing 5% skim milk (232100, Difco, Detroit, MI) for 30 min. After incubation with 100 µL of serially diluted serum samples or supernatant for 1 h, plates were again washed and incubated with 100 µL of anti-rat IgG (H+L)-HRP conjugate (A110-105P, Bethyl Laboratories, Montgomery, TX) diluted 50,000-fold in TBS-T (50 mM Tris-HCl, pH 8.0) (T1503, Sigma-Aldrich), 150 mM NaCl (195-01663, Fujifilm Wako Pure Chemical), 0.05% v/v Tween 20) for 30 min. Plates were washed again as described above, developed using 100 µL of *o*-phenylenediamine (160-11022, Fujifilm Wako Pure Chemical) for 30 min, and stopped using 25 µL of 1 M H_2_SO_4_ (95626-06, Nacalai Tesque, Kyoto, Japan). Absorbance was measured at 492 nm by microplate reader Epoch2 (Bio Tek, Winooski, VT).

### Western blotting

Cells were washed in cold PBS and lysed in lysis buffer containing 20 mM Tris-HCl (pH 7.4), 150 mM NaCl, 0.1% sodium dodecyl sulfate (SDS), 1% sodium deoxycholate, 1% Triton X-100, 1 µg/mL aprotinin, and 1 µg/mL leupeptin. Lysates were centrifuged at 15,000 rpm for 5 min. Protein concentrations were estimated using the Bradford protein assay (5000006, Bio-Rad Laboratories, Hercules, CA) with bovine serum albumin as the standard. The cell lysate was incubated with or without PNGase F (P0704S, New England Biolabs, UK) at 37 °C for 1 h.

Proteins were resolved by SDS-polyacrylamide gel electrophoresis using 10% gels, followed by electrotransfer to polyvinylidene difluoride membranes (ISEQ00010, Millipore, Bedford, MA). The membranes were then blotted using primary antibodies, washed, then incubated with secondary antibodies. The membranes were washed, and the bound antibodies were detected using an enhanced chemiluminescence detection system (GERPN2209, Amersham, Buckinghamshire, UK). The anti-AMIGO2 primary antibodies used in this study were rTNK1A0012; sc-373699, Santa Cruz Biotechnology, Dallas, TX; LS-C404504, LifeSpan BioSciences, Seattle, WA; #36094, Signalway Antibody, College Park, MD; HPA054004, Sigma-Aldrich), and anti-β-actin (1:2000; A1978, Sigma-Aldrich). The secondary antibodies used in this study were as follows: anti-mouse IgG-HRP (PM009-7, MBL, Nagoya, Japan), anti-rabbit IgG-HRP (458, MBL, Nagoya, Japan), and goat anti-rat IgG-HRP pre-adsorbed (ab98425, Abcam, Cambridge, UK).

### Patient samples

Immunohistochemical analysis was performed using paraffin-embedded colorectal cancer samples from 173 patients who underwent proctocolectomies at Tottori University Hospital between January 2007 and December 2015. For 173 cases, excluding the samples that were no longer available, the same samples reported in the previously were used [11]. Clinicopathological findings were determined using the Japanese classification of colorectal carcinoma [16]. None of the patients had received radiotherapy, chemotherapy, or other medical interventions before surgery.

### Immunohistochemistry

Tissue samples were fixed in formalin and embedded in paraffin. Serial sections were cut at 4 μm, deparaffinized in xylene, and rehydrated using a graded alcohol series. For retrieval of AMIGO2, the sections were heated at 121℃ for 20 min in an autoclave in 10 mM citrate buffer (pH 6.0). The samples were incubated in 0.1% hydrogen peroxidase for 15 min to block endogenous peroxidases and in 10% normal goat serum (424041, Nichirei Biosciences, Tokyo, Japan) for 15 min to prevent non-specific antigen binding. The slides were subsequently incubated with primary antibodies (rTNK1A0012a) overnight at 4 °C, then incubated with goat anti-rat IgG-HRP for 20 min. Staining was visualized with diaminobenzidine (SK-4105, Vector Laboratories, Burlingame, CA), and the sections were counterstained with hematoxylin. The expression of AMIGO2 in CRC cells was evaluated in a blinded manner. In brief, five fields were chosen at random and examined at x 400 magnification. The intensity of immunoreactivity was bisected according to a previous report [11], and the staining intensity of the primary CRC was defined as low (< 30% staining intensity) and high (≥ 30% staining intensity).

### Statistical analysis

All statistical analyses were performed using SPSS version 25 software (IBM Japan, Tokyo, Japan). The χ^2^ tests were used to compare the clinicopathological characteristics of tumors with high and low AMIGO2 expression. Univariate and multivariate analyses for the identification of prognostic factors for overall survival were carried out using the Cox proportional hazard regression model, and identification of prognostic factors for liver metastases were carried out using the logistic regression model. Kaplan-Meier survival curves were plotted and compared using a generalized log-rank test. Statistical significance was set at *p* < 0.05. Where appropriate, data are expressed as mean ± SD.

## Results

### Evaluation of reactivity of monoclonal antibody (rTNK1A0012) against human AMIGO2 protein

We developed rat monoclonal antibodies against the extracellular domain of human AMIGO2 for use in clinical diagnosis (Additional file 1 shows flowchart of the experimental method for establishing the monoclonal antibody [see Additional file 1]). The monoclonal antibodies with high immunoglobulin titers were selected by enzyme-linked immunosorbent assay (ELISA) (An additional file shows this in more detail [see Additional file 2]). Of the 19 candidate antibodies, the monoclonal antibody clone rTNK1A0012 (rTNK mAb) was established to detect human AMIGO2 for further study.

**Fig. 1:**
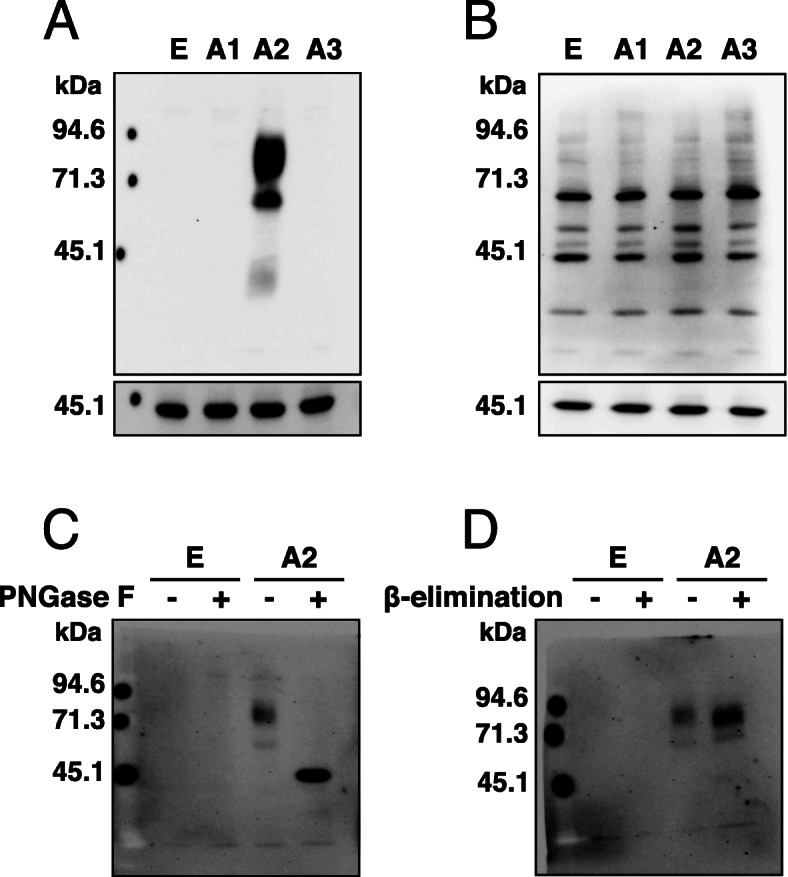
Specific detection of AMIGO2 by the monoclonal antibody rTNK1A0012. The large membrane was reacted with anti-AMIGO2 antibody, while the small membrane was reacted with an anti-β-actin antibody. **A** Cell lysates were prepared from HepG2 cells transfected with human AMIGO1 (A1), AMIGO2 (A2), AMIGO3 (A3), and an empty vector (E). Immunoblotting with rTNK1A0012 (rTNK mAb). **B** Immunoblotting with sc-373699 (sc mAb). **C** Cell lysates were treated with or without PNGase F and immunoblotted with rTNK mAb. **D** Cell lysates were treated with or without alkaline β-elimination and immunoblotted with rTNK mAb

**Fig. 2:**
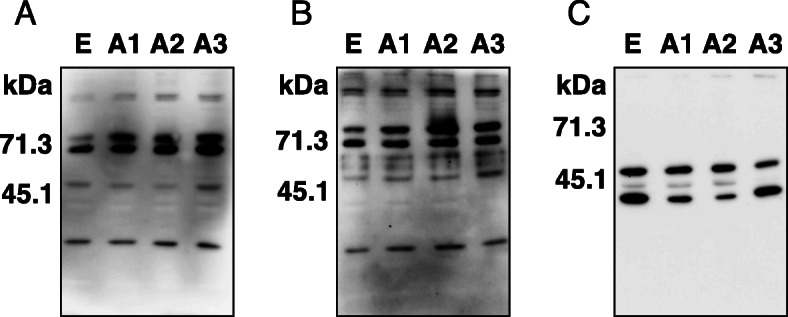
Detection of three types of AMIGO family molecules by commercially available antibodies. **A** The same cell lysates as shown in Fig. 1 was used. Lysates were prepared from HepG2 cells transfected with human AMIGO1 (A1), AMIGO2 (A2), AMIGO3 (A3), and an empty vector (E). Immunoblotting with LS-C404504 polyclonal antibody. **B** Immunoblotting with #36094 polyclonal antibody. **C** Immunoblotting with HPA054004 polyclonal antibody

To evaluate whether the rTNK mAb reacted specifically with human AMIGO2, HepG2 cells were transfected with a plasmid containing AMIGO1, AMIGO2, or AMIGO3 gene, and established stably expressing cell lines. Western blot analysis showed that rTNK mAb was reactive with AMIGO2 (HepG2-A2), while the rTNK mAb did not react with the other AMIGO family molecules; that is, AMIGO1 (HepG2-A1), AMIGO3 (HepG2-A3), or empty vector-transfected cells (HepG2-E) (Fig. 1 A). The rTNK mAb was found to be highly specific and sensitive to human AMIGO2. The same protein lysates obtained from the transfectants of the AMIGO family molecules were used to examine the specificity of the commercially available mouse anti-human AMIGO2 monoclonal antibody (sc-373699, sc mAb). On the other hands, the sc mAb cross-reacted with all types of AMIGO family molecules (Fig. 1B). Next, we investigated the specificity of three commercially available polyclonal antibodies (LS-C404504, #36094, and HPA054004). However, these antibodies were recognized as all AMIGO family molecules (Fig. 2).

In addition to the predicted molecular weight of human AMIGO2 based on the amino acid sequence of 58 kDa [22], multiple/diffuse bands suggest that the AMIGO2 protein may undergo a series of post-translational modifications such as glycosylation [23]. Since there are two main types of protein glycosylation, *N*-glycosylation and *O*-glycosylation [24], the peptide-*N*-glycosidase F (PNGase F), which cleaves *N*-linked oligosaccharides [25], was used first. Treatment of HepG2-A2 cell lysates with PNGase F caused a shift in electrophoretic mobility and resulted in conversion of the multiple/diffuse band to a single band (Fig. 1 C). In contrast, treatment of HepG2-A2 cell lysates with alkaline β-elimination, which releases *O*-linked glycans from *N*-glycosylated proteins [24], did not cause any shift in electrophoretic mobility (Fig. 1D). This indicates that the AMIGO2 protein is not *O*-glycosylated. It was clarified that the human AMIGO2 protein may undergo *N*-glycosylation as a post-translational modification but may not undergo *O*-glycosylation.

### Immunohistochemical detection of AMIGO2 expression by rTNK mAb and clinicopathologic risk factors

**Fig. 3:**
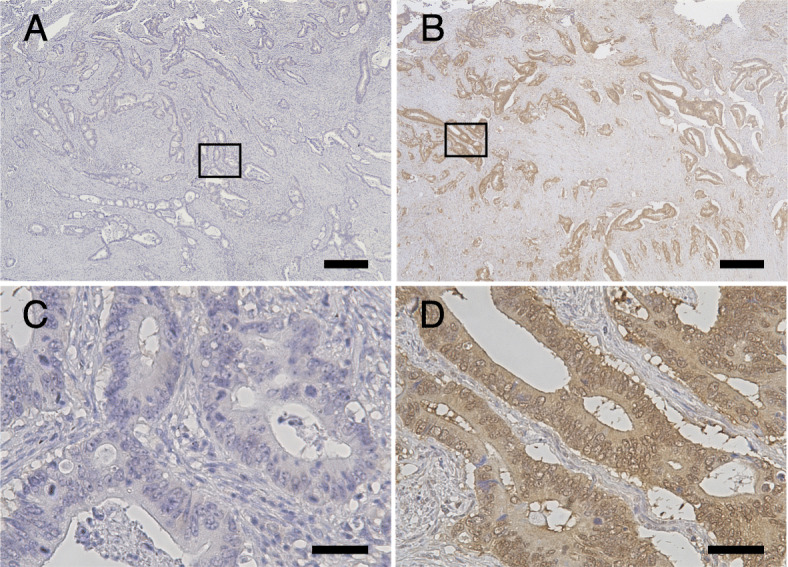
Immunohistochemical staining for AMIGO2 expression by the monoclonal antibody rTNK1A0012 in CRC tissues. AMIGO2 negative expression (**A** and **C**) and AMIGO2 high expression (**B** and **D**) are shown. The lower row are shown at higher magnification. Scale bars = 400 μm (**A** and **B**) and 100 μm (**C** and **D**)

The efficacy of the rTNK mAb against AMIGO2 was re-evaluated using the same CRC tissue as previously reported by immunohistochemical staining with the commercially available sc mA [11]. We performed immunohistochemistry of AMIGO2 in 173 CRC tissue samples. AMIGO2 was mainly expressed in tumor cells and was rarely expressed in the stroma (Fig. 3). AMIGO2 was located in both the cytoplasm and nucleus of the tumor cells (Fig. 3B and 3D). The expression of AMIGO2 was quantified using a visual grading system based on the extent of staining. The cut-off value for AMIGO2 expression was determined as the staining intensity of the primary CRC at 30%, as in a previous report [11], and the cases were divided into two groups: high and low expression. High expression was defined as an AMIGO2-positive tumor cell proportion of 30% or higher (≥ 30%, Fig. 3B and 3D). On the other hand, low expression was defined as less than 30% of positive tumor cells (< 30%, Fig. 3A and 3C).

**Fig. 4:**
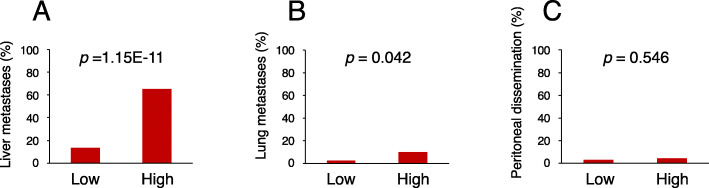
Relationship between AMIGO2 expression in primary colorectal cancer and metastatic site. A high expression of AMIGO2 was significantly associated with liver metastases (**A**) and lung metastases (**B**), but not with peritoneal dissemination (**C**), as calculated using the *X*^2^ test

Of the 173 tumor specimens evaluated, 28.3% (49/173) had AMIGO2 high expression, while 71.7% (124/173) had AMIGO2 low expression (Table 1). To evaluate the prognostic value of AMIGO2, we analyzed the correlations between clinicopathological variables and the expression of AMIGO2 in tumor tissues (Table 1; Fig. 4). A high expression of AMIGO2 (32/49; 65.3%) was more closely associated with liver metastasis than low AMIGO2 expression (17/124; 13.7%) (*p* = 1.15E-11; Fig. 4 A). Patients with high AMIGO2-expression tumors were found to be more likely to develop lung metastases (5/49; 10.2%) than patients with low expression tumors (3/124; 2.4%) (*p* = 0.042; Fig. 4B), whereas AMIGO2 expression was found to be independent of peritoneal dissemination (high 2/49, 4.1% vs. low 4/124, 3.2%: *p* = 0.546; Fig. 4 C). There was no significant correlation between AMIGO2 expression and age, sex, tumor location, tumor size, histological grade, depth of invasion, lymph node metastasis, lymphatic invasion, and vascular invasion (Table 1).

**Table 1:** AMIGO2 expression and clinicopathological factors affecting overall survival rate in 173 CRC patients

Variables	Total (n)	AMIGO2 expression		*p* value
		Low (*n* = 124)	High (*n* = 49)	
Age (years)				
< 70	87	64 (74%)	23 (26%)	0.580
≥ 70	86	60 (70%)	26 (30%)	
Sex				
male	95	66 (70%)	29 (30%)	0.478
female	78	58 (74%)	20 (26%)	
Tumor location				
colon	122	89 (73%)	33 (27%)	0.565
rectum	51	35 (69%)	16 (31%)	
Tumor size				
< 4.0 cm	68	47 (69%)	21 (31%)	0.548
≥ 4.0 cm	105	77 (73%)	28 (27%)	
Histological grade				
differentiated type^a^	157	110 (70%)	47 (30%)	0.115
undifferentiated type^b^	16	14 (88%)	2 (12%)	
Depth of invasion^c^				
T1/2	11	7 (64%)	4 (36%)	0.380
T3/4	162	117 (72%)	45 (28%)	
Lymph node metastasis				
absent	90	70 (78%)	20 (22%)	0.064
present	83	54 (65%)	29 (35%)	
Lymphatic invasion^d^				
ly0/1	65	48 (74%)	17 (26%)	0.623
ly2/3	108	76 (70%)	32 (30%)	
Vascular invasion^e^				
v0/1	97	75 (77%)	22 (23%)	0.050
v2/3	76	49 (65%)	27 (35%)	

^a^Well- and moderately differentiated adenocarcinoma

^b^Poorly differentiated adenocarcinoma, signet ring cell carcinoma, and mucinous carcinoma

^c^T1, lamina propria or submucosa invasion; T2, muscularis propria invasion; T3, subserosa invasion or within adventitia; T4, serosa penetration or adjacent organ invasion

^d^ly0, no invasion; ly1, minimal invasion; ly2, moderate invasion; ly3, severe invasion

^e^v0, no invasion; v1, minimal invasion; v2, moderate invasion; v3, severe invasion

### Improved correlation between AMIGO2 expression detected by rTNK1 mAb and liver metastasis and poor prognosis

In an univariate Cox regression analysis for different established risk factors for CRC, in addition to AMIGO2 expression (*p* = 0.005), age (*p* = 0.006) and vascular invasion (*p* = 0.019) were also associated with poor overall survival (Table 2). To compare the independent predictive value of AMIGO2 status for overall survival, a multivariate analysis with Cox’s proportional hazard regression model was performed. This analysis revealed that AMIGO2 status (*p* = 0.022), age (*p* = 0.007), and vascular invasion (*p* = 0.029) had an independent prognostic impact (Table 2). Of particular interest was whether AMIGO2 expression added any predictive value as a widely used prognostic factor for liver metastasis in CRC. Multivariate logistic regression analysis showed that the risk factors for liver metastases were significantly higher in patients with AMIGO2 high-expressing CRC than in those with AMIGO2 low-expressing tumors (*p* = 7.930E-10, Table 3).

**Table 2:** Univariate and multivariate analysis for overall survival in CRC patients (Cox proportional hazard regression model)

Variables^a^	Univariate analysis			Multivariate analysis		
	Hazard ratio	95% Confidence interval	*p* value	Hazard ratio	95% Confidence interval	*p* value
Age (< 70 vs. ≥ 70 years)	1.983	1.221–3.222	0.006	1.964	1.207–3.198	0.007
Sex (male vs. female)	0.669	0.413–1.083	0.102			
Tumor location (colon vs. rectum)	0.716	0.415–1.238	0.232			
Tumor size (< 4.0 vs. ≥ 4.0 cm)	1.285	0.789–2.092	0.314			
Histological grade (differentiated vs. undifferentiated)	1.303	0.524–3.237	0.569			
Depth of invasion (T1/2 vs. T3/4)	2.768	0.678–11.299	0.156			
Lymph node metastasis (absent vs. present)	1.163	0.725–1.864	0.532			
Lymphatic invasion (ly0/1 vs. ly2/3)	1.559	0.626–3.879	0.340			
Vascular invasion (v0/1 vs. v2/3)	1.755	1.096–2.810	0.019	1.698	1.055–2.734	0.029
AMIGO2 expression (low vs. high)	2.016	1.237–3.284	0.005	1.781	1.087–2.918	0.022

^a^Histological grade, depth of invasion, lymphatic invasion and vascular invasion were determined by the same grade as described in Table 1

**Table 3:** Univariate and multivariate analysis of factors affecting liver metastases in CRC patients (Logistic regression model)

Variables^a^	Univariate analysis			Multivariate analysis		
	Odds ratio	95% Confidence interval	*p* value	Odds ratio	95% Confidence interval	*p* value
Age (< 70 vs. ≥ 70 years)	0.764	0.393–1.484	0.427			
Sex (male vs. female)	0.485	0.242–0.970	0.041	0.443	0.197–0.998	0.049
Tumor location (colon vs. rectum)	0.734	0.345–1.561	0.422			
Tumor size (< 4.0 vs. ≥ 4.0 cm)	1.490	0.743–2.986	0.261			
Histological grade (differentiated vs. undifferentiated)	1.205	0.369–3.936	0.757			
Depth of invasion (T1/2 vs. T3/4)	1.839	0.383–8.834	0.447			
Lymph node metastasis (absent vs. present)	2.115	1.076–4.158	0.030			
Lymphatic invasion (ly0/1 vs. ly2/3)	1.532	0.756–3.101	0.236			
Vascular invasion (v0/1 vs. v2/3)	3.422	1.710–6.879	0.001			
AMIGO2 expression (low vs. high)	11.848	5.433–25.837	0.000^b^	12.254	5.511–27.247	0.000^c^

^a^Histological grade, depth of invasion, lymphatic invasion and vascular invasion were determined by the same grade as described in Table 1

^b^*p* = 5.146E-10

^c^*p* = 7.930E-10

**Fig. 5:**
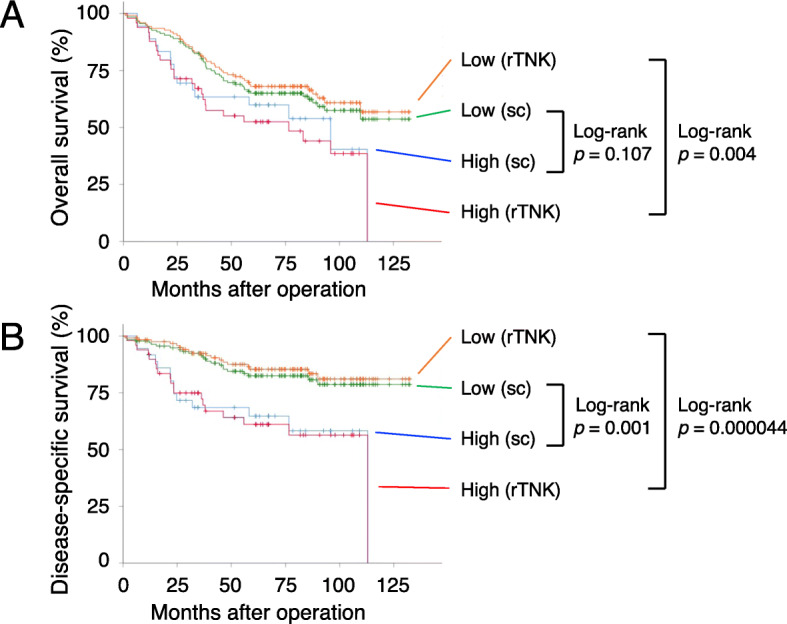
Comparison of two AMIGO2 antibodies in AMIGO2 expression and survival in CRC patients. Cumulative survival rates were assessed using the Kaplan-Meier plot method. Differences were analyzed using the log-rank test. Overall survival (**A**) and disease-specific survival (**B**) are shown

A Kaplan-Meier survival analysis was performed to compare which of the two anti-AMIGO2 monoclonal antibodies could more accurately determine the clinically worse outcome of CRC patients based on AMIGO2 expression levels. Overall survival tended to have a poor prognosis in patients with high AMIGO2 levels (Fig. 5 A). Most notably, in an analysis, rTNK mAb (*p* = 0.004), but not sc mAb (*p* = 0.107), predicted worse overall survival in patients with high AMIGO2 expression than in those with low AMIGO2 expression (Fig. 5 A). Moreover, a high expression of AMIGO2 resulted in short disease-specific survival, which is common to both antibodies, but rTNK mAb (*p* = 0.000044) was detected at a significantly higher level compared to sc mAb (*p* = 0.001; Fig. 5B).

The above findings showed that immunohistochemical detection of high AMIGO2 expression in CRC patients with rTNK mAb serves as a superior diagnostic biomarker and allows for prediction of poor prognoses by detecting liver metastasis compared to commercially available antibodies.

## Discussion

In this study, we produced a rat rTNK mAb specific for human AMIGO2, and confirmed that it detects AMIGO2 but does not cross-react with other AMIGO family molecules; that is, AMIGO1 and AMIGO3. By comparing the results previously reported for commercially available sc mAb with rTNK mAb using the same CRC tissues, the following five new facts and the usefulness of rTNK mAb were clarified: (i) the detection rate of liver metastases in CRC patients with high AMIGO2 expression in primary tumors improved from 47.5% for sc mAb [11] to 65.3% for rTNK mAb (Fig. 4 A); (ii) the association between AMIGO2 expression and liver metastasis by multivariate analysis resulted in predictability with sc mAb (*p* = 1.707E-5) [11]; but it was significantly predictable with rTNK mAb (*p* = 7.930E-10, Table 3); (iii) AMIGO2 expression and overall survival were not statistically significant when detected with sc mAb (*p* = 0.107), but was significantly correlated with worse prognosis when detected with rTNK mAb (*p* = 0.004, Fig. 5 A); (iv) in disease-specific survival, high AMIGO2 expression resulted in a poor prognosis, which can also be detected with sc mAb (*p* = 0.001), whereas it had a much higher correlation with rTNK mAb (*p* = 0.000044, Fig. 5B); and (v) factors affecting liver metastasis in multivariate logistic regression analysis using sc mAb were AMIGO2 expression (*p* = 1.707E-5), lymph node metastasis, vascular invasion, and sex [11]. In contrast, analysis using rTNK mAb eliminated the risk of lymph node metastasis and vascular invasion, leaving only AMIGO2 expression (*p* = 7.930E-10) and sex (*p* = 0.049). The above findings showed that by using rTNK mAb, AMIGO2 expression serves as a superior prognostic immunohistochemical biomarkers, especially for the detection of liver metastases and worse prognosis in CRC patients, compared to commercially available sc mAb.

Intriguingly, the use of rTNK mAb revealed that the AMIGO2 protein was *N*-glycosylated. Since it has been reported that the AMIGO1 protein, which is mainly expressed in the central nervous system, is *N*-glycosylated [26], the AMIGO family proteins may be included in those proteins in which half of all mammalian proteins are glycosylated as post-translational modifications [27]. In tumor tissues, abnormal glycosylation, that is, alterations to glycan epitopes, such as truncated *O*-glycans; altered *N*-glycan branching; increased sialylation; and fucosylation have been observed [23, 28–30]. These specific glycosylations have been shown to accelerate malignancies in tumor cells, such as signal transduction [31], growth [32], tumor immunity [33], epithelial-mesenchymal transition [34], motility [31], and metastasis [35], especially in CRC [36]. As a typical example of glycosylation-mediated liver metastasis in CRC patients, primary tumors expressing sialyl Lewis^X^ (sLe^X^) specifically bind or adhere to activated hepatic vascular E-selectin [31, 37–39]. We believed that CRC cells expressing AMIGO2 selectively form liver metastases by specifically binding to hepatic endothelial cells expressing AMIGO family molecules in a homophilic/heterophilic manner. To comprehensively understand the mechanism of liver metastasis due to AMIGO2 expression, it is necessary to examine how *N*-glycosylation of the AMIGO2 protein is associated with liver metastasis. Since there are eight putative *N*-glycosylation sites at 58, 104, 281, 288, 345, 373, 381, and 384 asparagine residues [22] in AMIGO2 that undergo *N*-glycosylation, we are going to investigate the degree of these *N*-glycosylation and liver metastatic activity.

The association between organ carcinogenesis and AMIGO2 expression has been reported in various types of cancer, including gastric cancer [13, 14], pancreatic cancer [20], colorectal cancer [11, 19], breast cancer [12], ovarian cancer [17], endometrial cancer [21], melanoma [15, 16, 40], and pituitary neuroendocrine tumors [18]. Since AMIGO2 expression is involved not only in tumor development but also in tumor progression including acquisition of metastasis, AMIGO2 may be a new molecule that controls cancer stem cell-like function. Elucidating the relationship between AMIGO2 expression and organ carcinogenesis and its malignant tumor progression not only improves the prognosis of cancer patients, but also determines target molecules for treatment and prevention. To proceed with these studies, it is conceivable to make great use of the specific antibody produced in this study.

In conclusion, we succeeded in establishing a monoclonal antibody that specifically recognizes human AMIGO2 and demonstrated its clinical applicability by immunohistochemistry. Pathological diagnosis by AMIGO2 expression using the rTNK mAb for surgically resected or biopsied primary CRC may be a promising new evaluation tool for predicting liver metastasis and poor prognosis in CRC patients.

## Supplementary information

Additional file 1. Flowchart of the experimental method for establishing a monoclonal antibody specific for human AMIGO2.

Additional file 2. The monoclonal antibodies with high immunoglobulin titers were selected by enzyme-linked immunosorbent assay (ELISA). From December 25, 2017 to January 22, 2018.

## Data Availability

All data generated or analyzed during this study are included in this published article.

## Abbreviations

AMIGO: amphoterin-induced gene and open reading frame
CRC: colorectal cancer
mAb: monoclonal antibody
sc mAb: anti-AMIGO2 mouse monoclonal antibody clone sc-373699
rTNK mAb: anti-AMIGO2 rat monoclonal antibody clone rTNK1A0012
