# Supplementary material for: Establishment of an antibody specific for AMIGO2 improves immunohistochemical evaluation of liver metastases and clinical outcomes in patients with colorectal cancer
Source: Diagn Pathol. 2022 Jan 30;17:16. doi: 10.1186/s13000-021-01176-2 (PMC8802484; doi:10.1186/s13000-021-01176-2)
Supplement: Supplementary file 1 — Flowchart of the experimental method for establishing a monoclonal antibody specific for human AMIGO2. [file 13000_2021_1176_MOESM1_ESM.pdf]

Additional File 1

Vector construction

Subcloning of AMIGO2 DNA fragment into pET-32(+) vector (pET32b-AMIGO2-EX protein)

↓

Elimination of leucine-rich repeat sequences (Trx-AMIGO2-Ig protein)

↓

Subcloning into pGEX-6P-1 vector (GST-AMIGO2-Ig fusion protein)

↓

Subcloning into pGEX-MCS-His vector (Trx-AMIGO2-Ig fusion protein)

Protein purification

Transformation of *E. coli* Rosseta-gami B pLysS strain by vectors

↓

Induction of protein by isopropyl-β-D (-)-thiogalactopyranoside

↓

Solubilization with guanidine hydrochloride & glutathione (redox form)

↓

Purification by Ni-NTA colum and dialysing

Immunization

Wister rats were i.p. injected with AMIGO2 protein with adjuvant every two weeks (prime & boost injections)

↓

AMIGO2 protein without adjuvant was injected i.v. (final boost)

Hybridoma generation and screening

Cell fusion of immunized rat spleen or lymph node cells with myeloma cells

↓

Selection of hybridoma clones producing AMIGO2 mAbs by ELISA

↓

Establishment of AMIGO2-specific mAb-producing hybridoma (rTNK1A0012)
