# Supplementary material for: Establishment of an antibody specific for AMIGO2 improves immunohistochemical evaluation of liver metastases and clinical outcomes in patients with colorectal cancer
Source: Diagn Pathol. 2022 Jan 30;17:16. doi: 10.1186/s13000-021-01176-2 (PMC8802484; doi:10.1186/s13000-021-01176-2)

The monoclonal antibodies with high immunoglobulin titers were selected by enzyme-linked immunosorbent assay (ELISA)

	Negative control	Trx-AMIGO2-Ig	GST-AMIGO2-Ig	Data acquisition date
TNK1A001	0.049	0.092	0.271	20171225
TNK1A004	0.046	0.128	0.19	20180122
TNK1A005	0.046	0.338	0.333	20180122
TNK1A007	0.044	0.071	0.089	20180122
TNK1A008	0.047	0.128	0.128	20180122
TNK1A009	0.044	0.165	0.192	20180122
TNK1A011	0.047	0.657	0.821	20180122
TNK1A012	0.044	0.425	0.677	20180122
TNK1A013	0.047	0.112	0.078	20180122
TNK1A016	0.05	0.118	0.106	20180122
TNK1A017	0.045	0.41	0.402	20180122
TNK1A018	0.046	0.075	0.08	20180122
TNK1A019	0.049	0.078	0.076	20180122
TNK1A022	0.05	0.476	0.407	20180222
TNK1A023	0.052	0.613	0.487	20180222
TNK1A024	0.043	0.199	0.286	20180222
TNK1A025	0.046	0.723	0.63	20180222
TNK1A026	0.048	0.555	0.521	20180222
TNK1A027	0.047	0.546	0.61	20180222

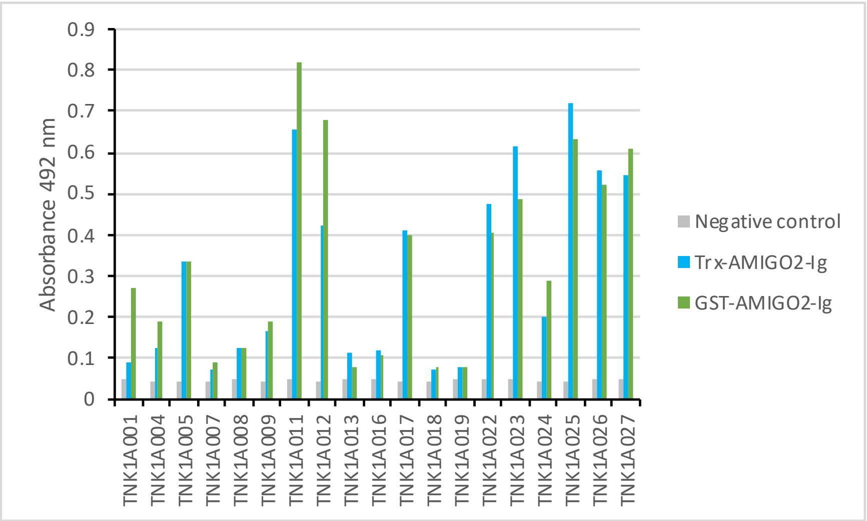

Supplement: Supplementary file 2 — The monoclonal antibodies with high immunoglobulin titers were selected by enzyme-linked immunosorbent assay (ELISA). From December 25, 2017 to January 22, 2018. [file 13000_2021_1176_MOESM2_ESM.pdf]
